# A comparison of three different diabetes screening methods among dental patients in Turkey

**DOI:** 10.12669/pjms.301.4238

**Published:** 2014

**Authors:** Rahsan Cevik Akyil, Ozkan Miloglu, Nermin Olgun, Ibrahim Sevki Bayrakdar

**Affiliations:** 1 Rahsan Cevik Akyil, Assistant Professor, Adnan Menderes University, Soke Health High School, Department of Internal Medicine Nursing, Aydin, Turkey.; 2 Dr. Ozkan Miloglu, Assistant Professor, Department of Oral Diagnosis and Radiology, Ataturk University, Faculty of Dentistry, Erzurum, Turkey.; 3 Prof. Dr. Nermin Olgun, Acibadem University, Faculty of Health Science, Department of Internal Medicine Nursing, Istanbul, Turkey.; 4 Dr. Ibrahim Sevki Bayrakdar, Research Assistant, Department of Oral Diagnosis and Radiology, Ataturk University, Faculty of Dentistry, Erzurum, Turkey.

**Keywords:** Diabetes, Undiagnosed diabetes, Type 2 diabetes, Screening methods

## Abstract

***Objective:*** This study was conducted to describe the frequency of diabetes in dental patients, and to compare three different screening methods: the random finger plasma glucose (RFPG) test, the Finnish diabetes risk score (FINDRISC) survey and a special clinical guideline developed for dental patients.

***Methods:*** The study design was cross-sectional, descriptive and comparative. The data were collected between August 2011 and February 2012. A total of 702 dental patients participated in this study. The screening tools were RFPG, FINDRISC and a clinical guideline. Data were analyzed using the Chi-squared test, the t test, analysis of variance, and the Pearson correlation test.

***Results:*** The frequency of diabetes was 8.3% for the participants. The prevalence of participants at risk for undiagnosed diabetes was 20.1% according to the RFPG test, 29.9% according to the FINDRISC, and 29.8% according to the clinical guideline. Correlation analysis showed a significant positive correlation between the screening methods (p<0.001 for each).

***Conclusion:*** The overall frequency of diabetes was 8.3%. It was found that the three screening methods used in this study were statistically similar. However, FINDRISC and clinical guideline as the questionnaire screening tools indicated a little larger group than RFPG with respect to diabetes risk.

## INTRODUCTION

According to an estimate by the World Health Organization (WHO), approximately 6.4% of the world’s adult population had diabetes in 2010. This value is expected to reach 7.8% by 2030,^1^ with type 2 diabetes accounting for about 90% to 95% of all diagnosed cases of diabetes,^2^ causing it to be recognized as a major global health problem.

In Turkey, previous studies reported that the prevalence of diabetes and prediabetes is 1.0 and 1.5%, respectively, in Turkey’s rural areas,^3^ while it reaches 6.4% and 11.6%,^5^^,^^6^ respectively, in the urban areas. According to the results of the Turkish Diabetes Epidemiology Study-I (TURDEP-I), the overall prevalence of diabetes was 7.2% in 1998 in Turkey.^7^ However, according to 2011 data, TURDEP-II reported that the prevalence of diagnosed diabetes is 9%.^8^

It is well known that type 2 diabetes may remain asymptomatic for several years and that about half of diabetic individuals are undiagnosed.^9^ Screening to improve early detection is one strategy that might permit early intervention, which is known to prevent or delay the onset of Type 2 diabetes and its complications.^10^^,^^11^

The Finnish diabetes risk score (FINDRISC) was developed to identify subjects at high risk for the future occurrence of type 2 diabetes.^12^ FINDRISC was originally developed in a prospective setting to identify persons at high risk of developing Type 2 diabetes.^13^

To identify a population at high risk of developing diabetes, a guideline developed by Li et al.^14^ is represented on the basis of data collection from the Third National Health and Nutrition Examination Survey (NHANES III)(1988-1994). The guideline has a sensitivity of 82.4% and a specificity of 52.8%. This instrument is inexpensive and noninvasive.

Random plasma glucose (RPG) remains the clinicians’ preferred screening method.^15^ Johnson et al.^15^ reported that screening strategies based on RPG balance sensitivity and speciﬁcity provide good results and minimize false-positive tests and costs. RPG screening for Type 2 diabetes has been reported to be worthwhile.^16^^,^^17^

This study was conducted to determine the frequency of diabetes in dental patients, and to compare three different screening methods: the random finger plasma glucose (RFPG) test, the Finnish diabetes risk score (FINDRISC), and a special clinical guideline developed for dental patients.

## METHODS

The research design was cross-sectional, descriptive and comparative. The data were collected from August 2011 to February 2012. A total of 702 dental patients who presented themselves to the oral diagnosis department of a dental faculty in Turkey were evaluated. Of the 702 participants, 58 had been previously diagnosed with diabetes (Type 1 or Type 2 diabetes). The population of the present study included randomly selected subjects. However, participants who were pregnant were excluded and so were participants less than 20 years old.

***Finnish Diabetes Risk Score (FINDRISC): ***The risk score form is a single page questionnaire.^12^ The total risk score is a simple sum of the individual scores, and values range from 0 to 26. The 10-year diabetes risk according to the final score is classified as "low risk" if less than 7, "slightly elevated risk" for 7-11, "moderate risk" for 12-14, "high risk" for 15-19 and "very high risk" for a score of 20 or more.

**Fig.1:**
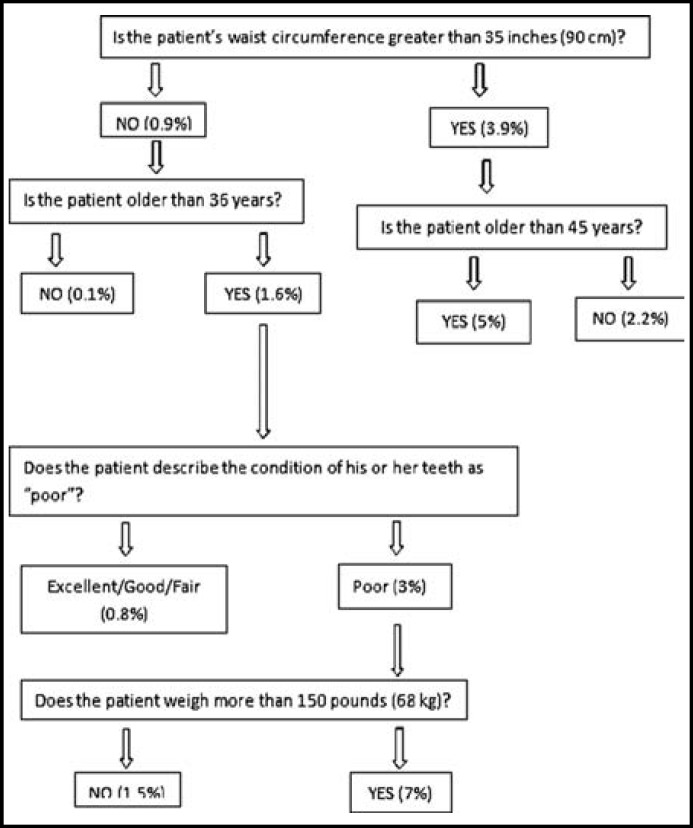
Flowchart of the clinical guideline (CG).

***The Clinical Guideline (CG): ***The clinical guidelines have been developed by Li et al.^14^ is composed of five steps, two initial steps that are part of the essential guide and three additional steps developed on the basis of recommendations made by the investigators who developed the guide. A flow chart of different questions and responses is also used to estimate the relative diabetes risk (Fig.1). Additionally, in the present study, the participants' periodontal status was also determined by physical examination and recorded.

***Random Finger Plasma Glucose (RFPG) Testing: ***The RFPG of these participants was measured using a regularly calibrated MediSense Optium machine (Abbott Laboratories, Doncaster, Australia). Single-use lancets were used; the first drop of blood was removed with a sterile tissue. 

In the present study, an RFPG of <100 mg/dL (5.6 mmol/L), both fasting and postprandial, was defined as a negative result. If RFPG was ≥100 mg/dL (5.6 mmol/L) fasting plasma glucose or ≥140 mg/dL (7.8 mmol/L) postprandial blood glucose, the result was accepted as a positive screening result. 

The study was approved by the Ethical Committee of the Health Sciences School of the university where the study was performed.

SPSS for Windows (version 11.5; SPSS, Chicago, IL) was used for data management and statistical analysis. Relations among the different groups and variables were analyzed with the χ^2 ^test or Fisher’s exact test, the t test and analysis of variance (ANOVA) where appropriate. Pearson correlation analysis was used to compare the screening methods.

## RESULTS

In the present study, the frequency of diabetes was 8.3% (Table-I). The distributions of the FINDRISC risk value means, the RFPG means and standard deviations and the "at risk" status according to CG are shown in Table-I. There was a statistically signiﬁcant difference between the age, sex, hypertension and coronary artery disease groups as to the distributions of FINDRISC risk value means (p˂0.001, p˂0.001, p˂0.001, and p˂0.05, respectively).

It was determined that there were statistically significant differences between age groups according to the CG’ results (p˂ 0.001). Additionally, there were statistically significant higher number of participants who were not at risk of diabetes in the nonsmoking, non-hypertensive, and the coronary artery disease-free groups (p˂ 0.001, p˂ 0.001, p˂ 0.05, respectively).

There was a statistically significant difference between the age groups according to the RFPG means obtained from both fasting and postprandial blood glucose (p˂ 0.001 for each). Additionally, the RFPG’ mean was higher in the group of participants with hypertension than in those who were non-hypertensive (p˂ 0.001).

**Table-I:** The prevalence of diabetes and the distributions of the FINDRISC, "at risk" status of CG, and RFPG means and standard deviations according to demographic features

			FINDRISC**			Clinical Guideline**					RFPG Test**					
		Prevalence of diabetes*				*Risk absent*		*Risk exist*			*Fasting blood glucose*			*Postprandial blood glucose *		
*Age group (years) *		*%*	*mean*	*SD*	*Significance*	*N*	*%*	*N*	*%*	*Significance*	*mean*	*SD*		*mean*	*SD*	*Significance*
	*20-29*	1.6	5.71	3.71	F =54.097 p < 0.001	245	100	-	-	χ^2^ = 404.496 p < 0.001	88.33	11.10	F =5.100 p < 0.001	102.80	18.00	F =10.290 p < 0.001
	*30-39*	1	7.75	3.55		97	98	2	2		92.66	17.93		113.80	13.30	
	*40-49*	12.9	9.96	3.91		85	66.4	43	33.6		94.31	16.41		124.50	29.06	
	*50-59*	11.1	11.81	4.02		8	8.3	88	91.7		100.39	19.96		130.38	65.72	
	*60-69*	34	11.22	3.23		6	17.1	29	82.9		96.00	16.56		137.11	22.93	
	*70-79*	7.4	12.36	3.35		4	16	21	84		95.52	14.87		151.00	20.78	
	*≥80*	11.1	14.62	3.05		7	43.8	9	56.3		91.92	7.30		178.00	21.21	
Sex																
	*Female*	9	9.25	4.63	t =4.417 p < 0.001	258	71.3	104	28.7	χ^2^ = 0.465 p > 0.05	93.24	15.45	t =-0.247 p > 0.05	118.35	27.48	t =1.718 p > 0.05
	*Male*	7.2	7.67	4.31		194	68.8	88	31.2		93.64	17.49		110.96	31.50	
Smoking																
	*Smoker*	10.1	7.92	3.95	t =-1.881 p > 0.05	118	83.1	24	16.9	χ^2^ = 14.515 p < 0.001	93.76	14.20	t =0.256 p > 0.05	109.78	23.61	t =-1.076 p > 0.05
	*Nonsmoker*	7.7	8.74	4.70		334	66.5	168	33.5		93.28	16.67		115.01	32.01	
Hypertension																
	*Yes*	26.2	13.39	2.92	t =12.276 p < 0.001	25	26.9	68	73.1	χ^2^ = 97.413 p < 0.001	97.18	12.73	t =2.173 p < 0.05	124.90	31.29	t =1.845 p > 0.05
	*No*	4.3	7.74	4.27		427	77.5	124	22.5		92.64	16.67		112.41	29.88	
Coronary artery disease																
	*Yes*	30.6	10.36	4.27	t =2.014 p < 0.05	13	52	12	48	χ^2^ = 4.111 p < 0.05	97.64	6.41	t =1.109 p > 0.05	119.75	16.58	t =0.573 p > 0.05
	*No*	7.1	8.49	4.55		439	70.9	180	29.1		93.21	16.43		113.49	30.62	
Total		8.3	8.56	4.55		452	70.2	192	29.8		93.38	16.17		113.73	30.20	

*; The data are given based on N=702, **; The data are given based on N=644

**Table-II:** The distribution of the RFPG results according to the FINDRISC risk scores, the CG risk level and threshold plasma glucose values (n=644).

			*Fasting blood glucose*				*Postprandial blood glucose *				*Total*	
			*N*	*mean*	*SD*	*Significance*	*N*	*mean*	*SD*	*Significance*	*N*	*%*
FINDRISC						*F=24.820 * *p ˂ 0.001*				*F=6.0700* *p ˂ 0.01*		
		Low	127	87.29	9.94		99	107.30	20.653		226	35.1
		Slightly elevated	144	90.69	12.63		81	116.73	22.094		225	34.9
		Moderate	100	95.86	16.77		22	120.77	61.279		122	18.9
		High	59	105.61	19.37		6	153.67	52.298		65	10.1
		Very high	6	125.33	39.05		-	-	-		6	0.9
		*Total*	436				208				644	100
CG												
		Risk absent	291	91.25	15.35	*t=-3.958 * *p ˂ 0.001*	161	107.09	20.467	*t=-6.421* *p ˂ 0.001*	452	70.2
		Risk exists	145	97.66	16.97		47	136.51	44.317		192	29.8
		*Total*	436				208				644	100
			*Fasting blood glucose*				*Postprandial blood glucose *					
*RFPG*			*N*	*%*			*N*	*%*				
	˂ 100 mg/dl		326	50.7		˂ 140 mg/dl	188	29.1				
		≥ 100 mg/dl	110	17		≥ 140 mg/dl	20	3.1				
		*Total*	436	67.7		*Total*	208	32.2			644	100

**Table-III:** Results of the Pearson correlation analysis between the screening methods used in the study (N=644).

	*RFPG*	*FINDRISC*	*CG*
	*r*	*r*	*r*
RFPG	-	0.175**	0.198**
FINDRISC	0.175**	-	0.466**
CG	0.198**	0.466**	-

r; Correlation Coefficient **; p ˂ 0.001

The distribution of the RFPG results according to the FINDRISC risk scores, the CG risk level and threshold values of the plasma glucose are shown in Table-II. According to FINDRISC, 29.9% of participants were at moderate, high and very high levels of risk, while 29.8% of participants were determined to have diabetes risk according to CG. The results indicated that 17% of participants had diabetes risk considering fasting blood glucose, 3.1% of participants had diabetes risk according to postprandial blood glucose value.

Pearson correlation analysis of FINDRISC, CG and RFPG data showed a positive correlation among all three screening methods (p <0.001 for all) (Table-III).

## DISCUSSION

TURDEP-I reported that the prevalence of diabetes was 7.2%.^7^ New data from TURDEP-II showed that, in 2011, the overall prevalence of known diabetes in Turkey was 9% and was 8.6% in eastern Turkey.^8^ The present study was conducted in Erzurum in eastern Turkey. Its results were similar to those reported by TURDEP-II.

The FINDRISC is considered a practical, non-invasive tool for screening for subjects at high risk for Type 2 diabetes in the population and in clinical practice.^13^ Two cutoff values of the FINDRISC were defined: subjects with score values in the range 7–14 were offered written information about a healthy lifestyle, whereas subjects scoring 15 or above were candidates for further testing for possible glucose abnormality and were referred for more intensive interventions.^18^ Therefore, several published studies reported that FINDRISC can be useful in determining undiagnosed diabetes risk at an early stage.^13^^,^^19^^-^^22^

In present study, the CG "at risk" characterization was only indicated if the highest risk level was attained in the final stage of the flow chart and either disturbed periodontal status or diabetes in the family history was present. Therefore, the CG was prepared with data from the subjects of NHANES III study, which included the subjects from the US. This CG also included groups defined in racial terms like Hispanic or African-American. While the original investigators included these racial categories in the flow chart, our patient universe did not include such subjects. Questions regarding racial classification were therefore excluded. This modification of the original questionnaire represents a limitation of our study.

Biochemical tests such as urine glucose and venous and capillary blood glucose under various conditions—fasting, at random, postprandial, or after a glucose load—have been used extensively for diabetes screening.^23^ ADA categorized the glucose levels of increased risk for diabetes as 100 mg/dL (5.6 mmol/L) to 125 mg/dL (6.9 mmol/L) for IFG (impaired fasting glucose), and 140 mg/dL (7.8 mmol/L) to 199 mg/dL (11 mmol/L) for IGT (impaired glucose tolerance).^24^ In the present study, RFPG’ threshold levels were defined according to the ADA’s recommendations.

The correlation analysis established the presence of a positive relation between all three screening methods. Although it has been reported that questionnaires tend to perform poorly for detecting undiagnosed type 2 diabetes, whereas biochemical tests perform better,^23^ the questionnaires indicate a little larger group with respect to diabetes risk in the present study. 

The present study was unable to precisely determine the efficiency of three screening tests, since a venous plasma glucose value was not available for all subjects. However it has been reported, that screening is warranted if it identifies patients with diabetes.^15^

In conclusion, the prevalence of diabetes was 8.3%. FINDRISC, a clinical guideline (CG) developed specifically for dental patients and RFPG were statistically similar to each other as screening method in dental patients. However, FINDRISC and clinical guideline as the questionnaire screening tools indicated a little larger group than RFPG with respect to diabetes risk.
